# Effect of Abdominal Massage with and without *Salvia officinalis* on Nausea and Vomiting in Patients with Cancer Undergoing Chemotherapy: A Randomized Clinical Trial

**DOI:** 10.1155/2021/9989228

**Published:** 2021-10-06

**Authors:** Farshid Rafiee Sarbijan Nasab, Parvin Mangolian Shahrbabaki, Mahlagha Dehghan, Haleh Tajadini, Hamideh Baniasadi, Sakineh Sabzevari

**Affiliations:** ^1^Nursing Research Center, Kerman University of Medical Sciences, Kerman, Iran; ^2^Department of Critical Care Nursing, Razi Faculty of Nursing and Midwifery, Kerman University of Medical Sciences, Kerman, Iran; ^3^Herbal and Traditional Medicine Research Center, Kerman University of Medical Sciences, Kerman, Iran; ^4^Department of Medical Surgical Nursing, Razi Faculty of Nursing and Midwifery, Kerman University of Medical Sciences, Kerman, Iran

## Abstract

**Objective:**

The aim of this study was to determine the effect of abdominal massage with and without *Salvia officinalis* on nausea and vomiting in patients with cancer undergoing chemotherapy.

**Methods:**

In this randomized clinical trial, 60 patients undergoing chemotherapy were placed in one of two intervention groups or in a control group. Abdominal massage with and without *Salvia officinalis* was performed for 15 minutes twice a day for 3 consecutive days by the patient's companion. The rate of nausea and vomiting was measured with a Visual Analog Scale.

**Results:**

Findings showed that immediately after the intervention, the mean score of nausea in abdominal massage with *Salvia officinalis* group was lower than that of the control group. The mean score of nausea was not different between abdominal massage and control groups. One week after the intervention, the mean score of nausea was not different among the three groups. In addition, the frequency of vomiting was not different among the three groups.

**Conclusion:**

Abdominal massage with/without *Salvia officinalis* as a complementary medicine has not considerable effect on reducing nausea and vomiting in patient with cancer undergoing chemotherapy. More studies are needed to achieve better and more accurate results.

## 1. Introduction

Chemotherapy is one of the main and most common treatments for patients with cancer [1, 2]. Among patients undergoing chemotherapy, complications such as nausea and vomiting are the most common, most painful, and most unpleasant side effects, with a prevalence of 54–96% of patients [3, 4].

Physical and psychological effects of chemotherapy in patients cause fear of starting chemotherapy and even resistance or rejection of anticancer treatment programs [5]. They also generate high cost expenses for patients and the health care system, such as prolonged hospitalization, increasing nursing and medical costs, and reducing patients' quality of life and performance [6, 7]. Even newer anticancer drugs are more toxic to the body, resulting in more nausea and vomiting, making their control more important and difficult than before.

According to the aforementioned studies and results, it seemed necessary to perform effective interventions to reduce chemotherapy-induced nausea and vomiting. It seems that consumption of antiemetic drugs, such as 5-hydroxytryptamine 3 (5-HT3) receptor antagonists, corticosteroids, neurokinin-1 (NK-1) receptor antagonists, serotonin receptor antagonists, dopamine antagonists, benzodiazepines, and neuroleptic drugs, used to reduce chemotherapy-induced nausea and vomiting only reduced vomiting and did not affect patients' nausea [7, 8]. Due to the limited effect and side effects of antinausea and vomiting drugs, one of the basic and low-risk measures is the use of complementary and alternative medicine [9]. Nonpharmacological methods to reduce chemotherapy-induced nausea and vomiting include abdominal massage, aromatherapy, thought distraction, acupuncture, relaxation, and music therapy [10, 11].

Abdominal massage is one of the complementary and alternative medicines for controlling chemotherapy-induced nausea and vomiting. Recently, nurses widely use abdominal massage to provide palliative care [11]. Abdominal massage with the help of mechanical and reflective methods increases intestine movements and changes abdominal pressure, followed by accelerating the passage of food along the gastrointestinal tract [12, 13]. This type of massage is noninvasive and leads to somatoautonomic reflex stimulation [14]. It, also, has few complications and can be performed by the patients themselves, their companions, or caregivers [3, 12].

Aromatherapy is the use of essential aromatic oils extracted from the roots, flowers, leaves, and stems of specific plants. Oils are quickly absorbed via the skin and into the bloodstream, and depending on the receptors they have, such as the brain and others, they can affect desire [15–17]. One of the most widely used drugs in traditional medicine is *Salvia officinalis*. It is used to treat colds, bronchitis, tuberculosis, and gastrointestinal diseases. In addition, it has anti-inflammatory, antibacterial, antifungal, antitumor, and antioxidant properties. This plant has these effects due to having oleacin acid and phenolic compounds that act like interferons in the body [15,17–20]. By releasing endorphin, massage with aromatic substance might physically relax and mentally calm the patients [15]. The results of a study by Sheikhi et al. indicated that therapeutic massage causes peace of mind and good psychological effects on health. It also reduces nausea and vomiting in patients with cancer. In addition, Mazlum et al.'s study showed that among the relaxation methods, Swedish-type massage therapy had the best results in reducing nausea and vomiting and also had a favorable psychological effect on the health of patients with cancer [21, 22]. However, other studies by Wang et al. and Hanai et al. on malignant patients with ascites and breast cancer reported that there was no significant difference between the participating groups, and abdominal massage did not affect the nausea [23, 24]. According to the literature review, there were limited studies on the effectiveness of abdominal massage in preventing gastric intolerance [11, 12]. The term gastric intolerance is frequently being used as a synonym for gastrointestinal dysfunction like nausea, vomiting, and constipation [25]. Therefore, according to the contradictory results and limited studies, the present study aimed to investigate the effectiveness of abdominal massage with and without *Salvia officinalis* on nausea and vomiting in patients with cancer undergoing chemotherapy.

## 2. Materials and Methods

### 2.1. Study Design and Setting

This randomized controlled clinical trial has been conducted in one of the oncology centers (47 beds) affiliated to the Kerman University of Medical Sciences, in Kerman, southeast of Iran.

### 2.2. Sample Size and Sampling

The subjects were selected using convenience sampling method from patients undergoing the same chemotherapy cycle in this center, and according to their documents, if they meet inclusion criteria, they could enroll to study. On the other hand, they assigned to a group by admission. They were allocated into three groups (two groups of intervention and one control group) by stratified block randomization method (stratum: sex). Labels A, B, and C (A = control, B = abdominal massage with aromatic substance, and C = abdominal massage without aromatic substance) were assigned to the groups using lottery, and the block size was six. Then, the randomization list was generated using free online software (https://www.sealedenvelope.com/simple-randomiser/v1/lists). The fourth author generated the randomization list, and the first author enrolled the participants and assigned them to the three groups. Due to the nature of the interventions, blinding was not possible.

Inclusion criteria were being older than 18 years [15], no coagulation disorder [7, 15], no history of migraine and chronic headache [15, 26], no history of being allergic to herbal medicines [15], not having Meniere's disease [27], not having respiratory problems [28], not being sensitive to aromatic substances [28, 29], not having a history of respiratory diseases, such as asthma, sinus disorders, and rhinitis [30, 31], not using aromatherapy for the patient for a week before the intervention [31], without colostomy, lack of acute abdominal surgery (such as intestinal obstruction, peritonitis, peptic ulcer, gastrointestinal bleeding, etc.), lack of local tumor or open wound in the abdomen, without chronic gastrointestinal diseases (such as ulcerative colitis, Crohn's, and irritable bowel syndrome), and lack of skin and respiratory allergies in the patient (the reason for choosing this criterion as inclusion and exclusion is that background issues do not interfere with findings).

**Figure 1:**
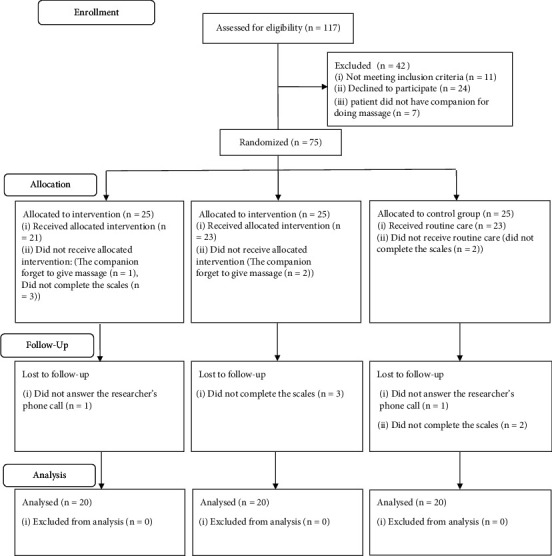
The study flow diagram.

Previous study was used for the estimation of sample size [32]. The confidence coefficient, the confidence interval, and the type II error were 95%, 1.96, and 20%, respectively (the study power = 80%). According to the three study groups, the sample size was adjusted, and the number of subjects needed for this study was 20 in each group (Figure 1).

### 2.3. Measurements

Data collection was performed by one nursing student who had not any information about the research process. Study tools included a demographic characteristics form, Visual Analog Scale (Mazlum et al.) for measuring nausea and vomiting, and to record the frequency of vomiting.

#### 2.3.1. Demographic Characteristics Form

The form contained information about the patient, including age, sex, marital status, job, income, educational level, duration of illness, type of disease, first date of chemotherapy, last date of chemotherapy, underlying diseases (such as gastrointestinal diseases, diabetes, and hypertension), medications received by the patient (narcotics and painkillers, chemotherapy drugs, gastrointestinal drugs), the patient's weight between two rounds of chemotherapy, and history of being allergic to herbal medicines.

#### 2.3.2. Visual Analog Scale (Mazlum et al.)

The VAS was used to measure the severity of nausea and vomiting. VAS was first introduced in 1972 by Clarcke and Spear [33]. Its score ranges from 0 to 10, the higher the score, the more severe the nausea and vomiting conditions. 0 showed no nausea and vomiting, 1 to 3 mild, 4 to 6 moderate, 7 to 9 severe, and 10 indicated very severe nausea and vomiting. This scale is a well-known tool for measuring the severity of nausea and vomiting. VAS is a standard tool, and its validity and reliability have been confirmed [34]. Patients' numbers of vomiting times in each day were recorded in another form.

### 2.4. Data Collection and Interventions

To collect data, first the demographic characteristics form was filled using the patients' medical documents and, if necessary, the patients and their companions. The subjects were then randomly assigned to two intervention groups or a control group. No intervention was performed in the control group, and the subjects were routinely cared for.

Abdominal Massage without Aromatic Substance Group (AM without AS Group)

In this group, the subjects received abdominal massage in addition to routine care. Before the massage, the patient's privacy was provided. The massage was performed half an hour before the meal time with an empty stomach due to the patient's greater comfort. The patient was placed in a supine position with the legs slightly bent at the knees, and the head at a 15- to 30-degrees angle. It should be noted that the patient's companion, after examining the patient's abdomen in terms of a mass and other contraindications to massage, would start the massage if there was no problem. Massage cause too little and rare problem, such as trauma, neurologic compromise, pain, dissection of arteries, and the like [35], that it is explained to patients, and they reported any unpleasant feeling. The massage started from the beginning of the ascending colon, in a clockwise direction, and continued towards the horizontal colon and finally to the end of the descending colon. The Swedish-type massage included strokes, effleurage, vibration, and kneading. The massage was performed 2 times a day for 15 minutes in 3 consecutive days for each patient at 8:30AM and 08:30PM. Massage hours were set so that the massage was performed at regular intervals and does not interfere with the patient's sleep hours [36]. In KMU Traditional Medicine school, one of the assistant professors of Traditional Medicine taught the first researcher how to perform the abdominal massage during 8 hours (four sessions) and approved his skill for educating abdominal massage to the patient's companion. The patient's companions were trained (during a 2-hour session), and a massage training brochure was provided. All massages were performed by a fixed companion. In addition, to ensure that the massages were performed, the time of the massage was reminded by sending a message.

Abdominal Massage with Aromatic Substance Group (AM with AS Group)

In this group, in addition to receiving routine care and massages like the aforementioned group, the abdominal massage was performed using 2 mL of *Salvia officinalis* aromatic substance with 100% concentration (made in Barij Essence Pharmaceutical Co., Kashan, Iran). *Salvia officinalis* is a shrub and a member of the mint family Lamiaceae and native to the Mediterranean region [19].

### 2.5. Data Analysis

Data were analyzed by SPSS 25. Descriptive statistics (frequency, percentage, mean, and standard deviation) were used to describe patients' demographic characteristics and clinical information. Mean and standard deviation was used to describe the nausea score. Frequency and percentage were used to describe vomiting. Chi-square test, Fisher's exact test, and ANOVA were used to examine the similarity of the three groups regarding the study variables. Regarding the confirmation of parametric conditions (normal distribution and equality of variances in the three groups), the repeated-measures ANOVA test was used to compare the changes in nausea score within and between the three groups at different times. The Bonferroni post hoc test was used for multiple comparisons. Significance level was considered 0.05.

### 2.6. Ethical Considerations

Kerman University of Medical Sciences approved the study protocol (No. IR.KMU.REC 1397.464). The study protocol was registered to the Iran RCT center (No. IRCT20141109019862N7). The researcher explained the intervention and obtained informed consent from the participants.

## 3. Results

The mean age of the AM with AS group was 55.30 ± 13.82 years, 54.45 ± 12.63 in the abdominal massage without aromatic substance group, and 52.40 ± 12.36 in the control group (*F* = 0.26, *P*=0.77). In terms of gender, 50% of the subjects in each group were female. The majority of the AM with AS group and the AM without AS group were homemaker/unemployed, and the majority of the control group subjects were employed (*P* > 0.05).

Between the three groups, there were no significant difference in terms of variables of weight, type of cancer, duration of cancer, duration of chemotherapy, other cancer treatments, and other diseases (Table 1). There was no significant difference between the three groups in terms of received drugs and chemotherapy regimen (Table 2).

According to Table 3, the results of the repeated-measures ANOVA showed that group-time interaction was not significant. However, each variable of group and time independently and significantly changed the mean score of nausea. In the AM with AS group, the mean score of nausea was 4.85 before, 1.80 immediately after, and 0.25 one week after the intervention, and nausea was significantly reduced after the intervention. In the AM without AS group, the mean score of nausea reduced from 4.80 before to 2.85 and 0.95 immediately after and one week after the intervention, respectively, and nausea was significantly reduced after the intervention. In the control group, the mean scores of nausea decreased from 4.60 before the intervention to 3.65 and 1.80 immediately after and one week after the intervention, respectively, and nausea was significantly reduced after the intervention. The results also showed that there was a significant difference between the three groups in terms of mean scores of nausea.

The results of a Bonferroni tests showed that the mean scores of nausea before the intervention were not significantly different between the three groups. While immediately after the intervention, the mean scores of nausea were significantly lower in the AM with AS group compared with the control group. The mean scores of nausea one week after the intervention did not differ significantly between the three groups (Table 4).

The results of post hoc Bonferroni tests to compare the changes in the mean scores of nausea at different times within each group are presented in Table 5.

Prior to the intervention, 35% of the subjects in the AM with AS group and 25% of the subjects in the massage without aromatic substance group and the control group had vomiting at least once a day. In this regard, there was no significant difference between the three groups (*χ*^2^ = 0.66; *P*=0.72). Immediately after the intervention, none of the subjects in the AM with AS group vomited, whereas 10% of the subjects in the AM without AS group and 25% in the control group had continued vomiting. Although the frequency of vomiting in the abdominal massage with and without aromatic substance groups were less than the control group, this difference was not statistically significant (Fisher's exact test = 5.73, *P*=0.06). One week after the intervention, none of the subjects experienced vomiting.

There were no reports of adverse effects about the interventions.

## 4. Discussion

The results of the present study showed that nausea severity only immediately after the intervention was significantly lower in the AM with AS group than in the control group. In addition, one week after the intervention, nausea severity was not different between the three groups, and it was at a moderate level.

The results of a review study by Sheikhi et al. indicated that therapeutic massage reduces nausea and vomiting in patients with cancer. In addition, Mazlum et al.'s study showed that among the relaxation methods, Swedish massage therapy had significantly reduced nausea and vomiting and also had a considerable psychological effect on the health of patients with cancer [21, 22]. The results of these two studies in reducing nausea severity were inconsistent with the results of the present study. In our study, using abdominal massage without aromatic substance had no extra effect on reducing nausea and vomiting in patients with cancer. The frequency of massage sessions, the tool for measuring nausea and vomiting, and the baseline severity of nausea and vomiting were different between our study and the mentioned studies. Therefore, these reasons may be the cause of different results in different studies.

However, the results of a study by Hanai et al. on 30 patients with breast cancer indicated that abdominal massage did not affect nausea [23]. The results of Hanai et al.'s study are in line with the present study. However, there are differences between Hanai et al.'s study and the present study. First, Hanai et al.'s study had two groups, that is, intervention and control, and no aromatic substance was used. Second, the sample size was different in the two studies, and only patients with breast cancer were included in Hanai et al.'s study. Third, there was a difference between the two studies in terms of interventions. Hanai et al. used a combination of abdominal massage and abdominal exercise. Fourth, the total time for massage and exercise for each patient was 5-6 minutes, and the patients were asked to repeat the intervention 10 times a day. Fifth, in Hanai et al.'s study, the patients were taught to perform the intervention themselves. Finally, Hanai et al. used Common Terminology Criteria for Adverse Events to measure patients' nausea.

Another study by Wang et al. on Eighty patients with cancer indicated that abdominal massage had no effect on patients' nausea [24]. The results of Wang et al.'s study were consistent with the results obtained from the abdominal massage group without aromatic substance in the present study and were inconsistent with the results obtained from the abdominal massage group with officinalis aromatic substance. It should be noted that there were differences between the two studies. In Wang et al.'s study, no aromatic substances were used in the intervention, and only abdominal massages were applied. In their study, abdominal massages were performed twice a day (morning and evening) for 15 minutes each time, and the intervention was repeated for 3 days for each patient. It should also be noted that in their study, massages were performed by a nurse. These differences in how the two studies were conducted might have led to differences in the results. The results of the present study and the reviewed studies showed that abdominal massage did not have a positive effect on reducing nausea in patients. But what is worth noticing is the effect of abdominal massage with aromatic substance on patients' nausea. According to studies on the effect of aromatic substance on reducing the occurrence of nausea in patients [37, 38], the results of the present study might be justified by the fact that just the inhalation of aromatic substance had reduced the occurrence of nausea in patients of the group of abdominal massage with aromatic substance. However, given that very limited studies have been found through literature review on the effect of abdominal massage on the occurrence of nausea in patients with cancer and other patients, it is not possible to speak with certainty about the effectiveness or ineffectiveness of this treatment. Future studies in this area could lead to more definitive and better results. In line the present results, Zorba and Ozdemir found that massage with aromatherapy can reduce chemotherapy adverse effects, such as vomiting and retching [39]. In addition, Izgu et al. reported that aromatherapy massage may be useful in the management of chemotherapy-induced peripheral neuropathic pain and fatigue [40]. However, some studies demonstrated that aromatherapy could not affect gastrointestinal reactions due to chemotherapy [41, 42]. Maybe using different aromatic substances in different studies is the reason of different results.

The results of the present study showed that the frequency of vomiting decreased immediately after the intervention in the AM with AS group and the AM without AS group decreased compared with before the intervention; but in the control group, there were no changes in the frequency of vomiting before and immediately after the intervention. Although the frequency of vomiting was lower in the massage with aromatic substance group and the AM without AS group than in the control group, this difference was not statistically significant. One week after the intervention, none of the subjects experienced vomiting.

The results of Hanai et al.'s study showed that there was no significant difference between the groups participating in the study in terms of vomiting and abdominal massage did not affect it [23]. Given that in the present study, despite the lack of statistical significance between the studied groups, we saw a decrease in the frequency of vomiting in the intervention groups and a positive effect of abdominal massage; therefore, this study was inconsistent with Hanai et al.'s study. One of the reasons for the inconsistency might be the difference between the two study populations. In Hanai et al.'s study, only patients with breast cancer were included. There was also a difference between the two studies in terms of conducting the study and performing the abdominal massage.

In Wang et al.'s meta-analysis on the effects of abdominal massage on Gastrointestinal function in patients in the intensive care unit, they had reviewed several databases. The results of 9 studies, which included 720 patients, indicated that abdominal massage was effective in reducing vomiting in patients [43]. According to the results of the present study and the positive effect of abdominal massage on reducing the frequency of vomiting, these two studies could be considered consistent. Uysal conducted a study on the effect of abdominal massage on residual stomach volume on 80 patients in the neurology department. He concluded that after the intervention, vomiting occurred in 10% of patients in the control group, and there was no occurrence of vomiting in patients of the intervention group. But this difference was not statistically significant [44]. These results were consistent with the results of the present study.

In another study by Uysal on the effect of abdominal massage administered by caregivers on gastric complications occurring in 100 patients with intermittent enteral feeding, the results showed that abdominal massage reduced vomiting in patients in the intervention group compared with the control group, and this reduction was statistically significant [26]. Since the results of the present study indicated the positive effect of abdominal massage on patients' vomiting, it could be said that the results of the two studies were consistent. However, since this reduction was not statistically significant in the present study, the results were inconsistent with Uysal's study. There were differences in terms of clinical and care status as well as the sample size between Uysal's study and the present study. In Uysal's study, there were two groups of participants (intervention and control), and the intervention group used only abdominal massage. Massage movements were also somewhat different in the two studies.

Reviewing the results of the above studies showed that abdominal massage could be effective in reducing the occurrence of vomiting in patients. New studies suggest that patients often seek medical attention that can be performed outside a clinic or hospital. These interventions include complementary and alternative methods. Massage therapy is one of the most widely used methods in cancer patients. It reduces stress hormones, such as cortisol, epinephrine, and norepinephrine, thereby reduces anxiety, nausea, vomiting, and relaxes the patients [45]. Given that vomiting is one of the most common and irritating side effects of chemotherapy, using massage therapy to improve patients' well-being and reduce the side effects of chemotherapy could be effective and useful.

Abdominal massage can increase intestine movements and changes abdominal pressure. Probably, abdominal massage through the somatoautonomic reflex may produce anal waves. On the other hand, compression of the abdomen can directly stimulate the intestinal pressure receptors, and this stimulation may initiate anal contractions. These changes can relieve gastrointestinal problems, such as nausea, vomiting, and constipation, and improve intestinal muscle tone [12, 44]. Usually, when aromatic oils are used with massage, these oils are absorbed through the skin with rhythmic pressures and then enter the bloodstream. In fact, the combination of oils with massage facilitates absorption of oils through the skin. It is gradually absorbed through the skin barrier for 10–40 minutes and after absorption, its therapeutic effects appears, including sedation, analgesia, antispasmodics and cramps, vascular vasodilation, and raising skin temperature [15, 17].

The present study had its limitations. Taking medications that affect the movements and function of the gastrointestinal tract (such as calcium pump inhibitors and dopamine) by patient was one of the limitations, which we tried to put a number of drugs affecting the movements of the gastrointestinal tract in the inclusion criteria, and the patient's medication regimen was also checked. Due to the difference in body mass in different people, the effect of massage could be different. Given this issue, studied samples' weight in all three groups were measured. Considering the number of sample sizes and the fact that sampling has been performed from a single city, generalizations should be made with caution. In addition, one of the limitations is using weight instead of abdominal waist circumference measurement. Abdominal waist circumference may indicate a better gastrointestinal function than weight. Distention is one of the adverse effects of abnormal gastrointestinal function that increases abdominal waist circumference. Finally, chemotherapy-induced nausea is a multifactorial symptom and abdominal massage, based on available theories reduce gastric residual volume. Therefore, it is suggested that future studies use bundle of interventions to target different causes of chemotherapy-induced nausea.

## 5. Conclusion

Abdominal massage with/without *Salvia officinalis* as a complementary medicine has not considerable effect on reducing nausea and vomiting in patient with cancer undergoing chemotherapy. Although abdominal massage with/without aromatic substance is a simple, inexpensive, and applicable treatment that its positive effects had demonstrated in many studies, more researches are needed to confirm its effect on reducing gastrointestinal intolerance in patient with cancer.

## Figures and Tables

**Table 1:** Description of participations/clinical information in three study groups.

Variables	Group							
	The AM with AS group		The AM without AS group		The control group		Statistical analysis	*P* value
	Mean	SD	Mean	SD	Mean	SD		
Duration of cancer (months)	8.95	6.50	8.25	4.55	8.25	6.73	*F* = 0.09	0.91
Duration of chemotherapy (months)	5.70	4.11	6.90	4.61	6.70	6.21	*F* = 0.32	0.72
Weight (kg)	62.60	13.57	63.75	11.06	63.95	12.90	*F* = 0.07	0.94
	*n*	%	*n*	%	*n*	%		
Type of cancer								
Breast	7	35.0	4	20.0	4	20.0	*χ* ^2^ = 8.14	0.62
Blood	2	10.0	2	10.0	2	10.0		
Respiratory tract	2	10.0	4	20.0	2	10.0		
Gastrointestinal	4	20.0	4	20.0	5	25.0		
Reproductive	2	10.0	4	20.0	1	5.0		
Others^*∗*^	3	15.0	1	5.0	6	30.0		
Other cancer treatments								
No	7	35.0	9	45.0	10	50.0	Fisher's exact test = 4.22	0.65
Surgery	8	40.0	8	40.0	9	45.0		
Radiotherapy	2	10.0	2	10.0	0	0		
Surgery and radiotherapy	3	15.0	1	5.0	1	5.0		
Other underlying diseases^*∗∗*^								
No	18	90.0	19	95.0	19	95.0	*χ* ^2^ = 0.54	0.76
Yes	2	10.0	1	5.0	1	5.0		

^*∗*^Melanoma, lymphoma, and pancreatic cancer. ^*∗∗*^Diabetes and hypertension. AM: abdominal massage; AS: aromatic substance; SD: standard deviation; *F*: analysis of variance.

**Table 2:** Comparison of the drug type received by the patient in the three study groups.

Variables	Group							
	The AM with AS group		The AM without AS group		The control group		Statistical analysis	*P* value
	n	%	n	%	n	%		
Opium								
Yes	4	20.0	5	25.0	5	25.0	*χ* ^2^ = 0.19	0.91
No	16	80.0	15	75.0	15	75.0		
Painkillers								
Yes	9	45.0	8	40.0	8	40.0	*χ* ^2^ = 0.14	0.93
No	11	55.0	12	60.0	12	60.0		
Digestive								
Yes	9	45.0	9	45.0	5	25.0	*χ* ^2^ = 2.26	0.32
No	11	55.0	11	55.0	15	75.0		
Carboplatin								
Yes	2	10.0	2	10.0	1	5.0	*χ* ^2^ = 0.44	0.80
No	18	90.0	18	90.0	19	95.0		
Cisplatin								
Yes	4	20.0	3	15.0	3	15.0	*χ* ^2^ = 0.24	0.89
No	16	80.0	17	85.0	17	85.0		
Doxorubicin								
Yes	1	5.0	0	0.0	3	15.0	Fisher's exact test = 3.11	0.31
No	19	95.0	20	100.0	17	85.0		
Endoxan								
Yes	3	15.0	2	10.0	6	30.0	*χ* ^2^ = 2.89	0.24
No	17	85.0	18	90.0	14	70.0		
Etoposide								
Yes	2	10.0	1	5.0	1	5.0	*χ* ^2^ = 0.54	0.76
No	18	90.0	19	95.0	19	95.0		
Fluorouracil								
Yes	5	25.0	6	30.0	4	20.0	*χ* ^2^ = 0.53	0.77
No	15	75.0	14	70.0	16	80.0		
Gemcitabine								
Yes	2	10.0	3	15.0	1	5.0	*χ* ^2^ = 1.11	0.86
No	18	90.0	17	85.0	19	95.0		
Herceptin								
Yes	3	15.0	0	0.0	1	5.0	Fisher's exact test = 3.11	0.31
No	17	85.0	20	100.0	19	95.0		
Irinotecan								
Yes	1	5.0	2	10.0	1	5.0	*χ* ^2^ = 0.54	0.76
No	19	95.0	18	90.0	19	95.0		
Leucovorin								
Yes	2	10.0	2	10.0	3	15.0	*χ* ^2^ = 0.32	0.85
No	18	90.0	18	90.0	17	85.0		
Oxaliplatin								
Yes	1	5.0	1	5.0	2	10.0	*χ* ^2^ = 0.54	0.76
No	19	95.0	19	95.0	18	90.0		
Paclitaxel								
Yes	3	15.0	1	5.0	2	10.0	*χ* ^2^ = 1.11	0.57
No	17	85.0	19	95.0	18	90.0		
Taxotere								
Yes	2	10.0	1	5.0	1	5.0	*χ* ^2^ = 0.54	0.76
No	18	90.0	19	95.0	19	95.0		
Zoladex								
Yes	0	0.0	3	15.0	1	5.0	Fisher's exact test = 3.11	0.31
No	20	100.0	17	85.0	19	95.0		
Other chemotherapy drugs^*∗*^								
Yes	2	10.0	6	30.0	8	40.0	*χ* ^2^ = 4.77	0.09
No	18	90.0	14	70.0	12	60.0		

^*∗*^Methotrexate and bleomycin. AM: abdominal massage; AS: aromatic substance.

**Table 3:** Comparison of the mean scores of nauseas at different times between the three groups.

Variables	Group					
	The AM with AS group		The AM without AS group		The control group	
	Mean	SD	Mean	SD	Mean	SD
Before the intervention	4.85	2.62	4.80	2.75	4.60	2.46
Immediately after the intervention	1.80	1.67	2.85	2.01	3.65	1.60
One week after the intervention	0.25	0.44	0.95	1.05	1.80	1.10
The source of change	Sums of squares		Degrees of freedom	*F*	*P* value	Eta^2^
Group	30.28		2	4.0	0.02	0.045
Time	337.34		2	44.52	<0.001	0.34
Group*∗*time	24.49		4	1.62	0.17	0.04
Error	647.80		171			

AM: abdominal massage; AS: aromatic substance; SD: standard deviation; *F*: repeated-measures analysis of variance.

**Table 4:** Results of post hoc Bonferroni tests on the comparison of changes in the mean scores of nausea at different times between the three groups.

Time	Group (I)	Group (J)	Mean of changes (I−J)	Standard Error	*P* value
Before the intervention	The AM with AS group	The AM without AS group	0.05	0.62	>0.99
		The control group	0.25	0.62	>0.99
	The AM without AS group	The control group	0.20	0.62	>0.99

Immediately after the intervention	The AM with AS group	The AM without AS group	−1.05	0.62	0.27
		The control group	−1.85	0.62	0.009
	The AM without AS group	The control group	−0.8	0.62	0.59

One week after the intervention	The AM with AS group	The AM without AS group	−0.75	0.62	0.67
		The control group	−1.40	0.62	0.07
	The AM without AS group	The control group	−0.65	0.62	0.88

AM: abdominal massage; AS: aromatic substance.

**Table 5:** Results of post hoc Bonferroni tests to compare the changes in the mean scores of nausea at different times within each group.

Group	Time (I)	Time (J)	Mean of changes (I−J)	Standard error	*P* value
The AM with AS group	Immediately after the intervention	Before the intervention	−3.05	0.62	<0.001
	One week after the intervention	Before the intervention	−4.15	0.62	<0.001
		Immediately after the intervention	−1.1	0.62	0.23

The AM without AS group	Immediately after the intervention	Before the intervention	−1.95	0.62	0.005
	One week after the intervention	Before the intervention	−3.35	0.62	<0.001
		Immediately after the intervention	−1.40	0.62	0.07

The control group	Immediately after the intervention	Before the intervention	−0.95	0.62	0.37
	One week after the intervention	Before the intervention	−2.5	0.62	<0.001
		Immediately after the intervention	−1.5	0.62	0.04

AM: abdominal massage; AS: aromatic substance.

## Data Availability

The datasets used and/or analyzed during the current study are available from the corresponding author on reasonable request.
